# Diabetic Nephropathy, Retinopathy, and Functional Hypogonadism in a Patient with MODY10: A Case Report

**DOI:** 10.3390/medicina60050830

**Published:** 2024-05-18

**Authors:** Rossana Ruiz-Urbaez, Mariela Viviana Villagómez-Estrada, Carlos Reyes-Silva, Darlyng Quishpe-López, David Males-Maldonado, Jorge Salazar-Vega, Enrique Gea-Izquierdo

**Affiliations:** 1 Unit of Endocrinology and Diabetes, Eugenio Espejo Hospital, Quito 170403, Ecuador; 2 Unit of Endocrinology, Dr. José Eleuterio González Hospital, Monterrey 64460, Mexico; 3 Unit of Genetic, Eugenio Espejo Hospital, Quito 170403, Ecuador; 4 Faculty of Medicine, Pontifical Catholic University of Ecuador, Quito 170143, Ecuador; 5 Department of Medical Specialties and Public Health, Rey Juan Carlos University, 28922 Madrid, Spain; 6 María Zambrano Program-European Union, Rey Juan Carlos University, 28922 Madrid, Spain

**Keywords:** maturity-onset diabetes of the young (MODY), INS gene, nephropathy, retinopathy, hypogonadism

## Abstract

(1) *Background and objectives*: Maturity-onset diabetes of the young (MODY) is a group of diabetes caused by gene defects related to insulin secretion. MODY1, MODY2, and MODY3 are the most common and account for approximately 80% of all cases. Other types are relatively rare. This study describes the clinical, analytical, and genetic characteristics of a patient with MODY10, and diabetic nephropathy, retinopathy, and functional hypogonadism diagnosis. (2) *Materials and methods*: A clinical case was analyzed and whole exome generation sequencing (WES) was used to detect mutations related to a monogenic variant. (3) *Results*: A seventeen-year-old male patient, who was diagnosed with apparent type 1 diabetes at the age of eight was started with insulin therapy. He came to the emergency room with glycemic decompensation, facial, and lower limb edema. During his evaluation, he had near-nephrotic range proteinuria of 2902 mg/24 h, a kidney ultrasound showing mild pyelocalyceal dilation, proliferative diabetic retinopathy, and was also diagnosed with functional hypogonadotropic hypogonadism. These comorbidities improved with adequate glycemic control. WES showed missense variant c.94G>A (p.Gly32Ser) in the INS gene, according to Clinvar corresponding to MODY10. It was a “de novo” variant not reported in his parents. (4) *Conclusions*: Monogenic diabetes (MD) is rare and MODY10 is among the less frequent types. MODY should be suspected in patients with type 1 phenotype with negative autoimmunity even in the absence of a family history of diabetes. To the best of our knowledge, we present here the first patient with these phenotypic traits of MODY10 reported in Latin America.

## 1. Introduction

Diabetes mellitus is a disease caused by an alteration of metabolism, characterized by an increase in the amount of blood glucose and the appearance of microvascular and cardiovascular complications. This, in turn substantially increases damage to other organs, and the mortality associated with the disease. It reduces the quality of life of the affected people and affects 5–10% of the general population [1].

Diabetes and especially poorly controlled diabetes can cause damage to the kidneys with diabetic nephropathy (DN) [2]. DN is the leading cause of chronic kidney disease or end-stage renal disease (ESRD), affecting around 700 million people. It disproportionately affects those who are socially disadvantaged. Half of all patients in the U.S. with ESRD have diabetes reported as the primary etiologic cause. DN is an independent predictor of cardiovascular disease (CVD) and mortality in patients with diabetes. Therefore, early diagnosis and treatment are extremely important for the prevention of not only the DN progression but also CVD [3]. Diabetic retinopathy (DR) is a leading cause of new cases of visual loss among working-age worldwide. A quarter of the diabetic population is affected by any level of DR and 5% have a severe degree. Furthermore, DR is responsible for 10% of new cases of blindness every year, with a risk of development 25 times higher about this complication compared to the rest [4].

Monogenic diabetes (MD) is a rare disorder that results from mutations in a single gene. On the other hand, the most common types of diabetes—type 1 and type 2—are caused by several genes (and in diabetes type 2, lifestyle factors, such as obesity). In most cases, diabetes is inherited. MD includes a variety of conditions characterized by early-onset diabetes, such as neonatal diabetes, maturity-onset diabetes of the young (MODY), and diabetes associated syndromes [5]. MD is mostly caused by impaired function or development of pancreatic islets, with defective insulin secretion in the absence of obesity [6]. The most prevalent form of MD is MODY, a collection of clinically heterogeneous inherited disorders of non-autoimmune diabetes mellitus (antibody-negative), that present at a young age of onset [7]. The disease can be mild or severe, depending on the gene involved. More than 20 subtypes of MODY have been identified to date, estimated to account for 0.5 a 5% of all diabetes cases [6], though these estimates likely underrepresent the true prevalence and there is a marked scarcity of data in Latin America [8]. It generally occurs in children or adolescents, but sometimes it is not detected until they are adults. Up to 3.5% of cases are diagnosed before 30 years of age [9], but most of these patients are misdiagnosed as having either type 1 or type 2 diabetes, the most prevalent being deficiencies of glucokinase (GCK) hepatocyte nuclear factor-1 alpha (HNF1A)), and hepatocyte nuclear factor-4 alpha (HNF4A), formerly known as MODY2, MODY3, MODY1. Subtype 10 (INS-MODY) is a very rare autosomal dominant condition caused by a mutation of the INS gene, located in chromosome 11p15.5, which encodes insulin [10]. It was first described in 2008 by Edghill et al. [11].

Hypogonadism is defined as testosterone deficiency with associated signs or symptoms, deficiency in sperm production, or both. It may be due to a disorder of the testicles (primary hypogonadism) or the hypothalamic–pituitary axis (secondary hypogonadism). Hypogonadotropic hypogonadism is a clinical entity caused by hypothalamic-pituitary insufficiency that results in deficient secretion of gonadotropins. Functional hypogonadism is defined as having low testosterone levels, being potentially reversible, and without an intrinsic structural anomaly of the hypothalamic–pituitary–gonadal axis, with obesity and poorly controlled diabetes mellitus being its most common causes [12].

The aim of this study Is to present an uncommon clinical case of MODY10 with retinal, kidney involvement, and functional hypogonadotropic hypogonadism.

## 2. Detailed Case Description

**Figure 1:**
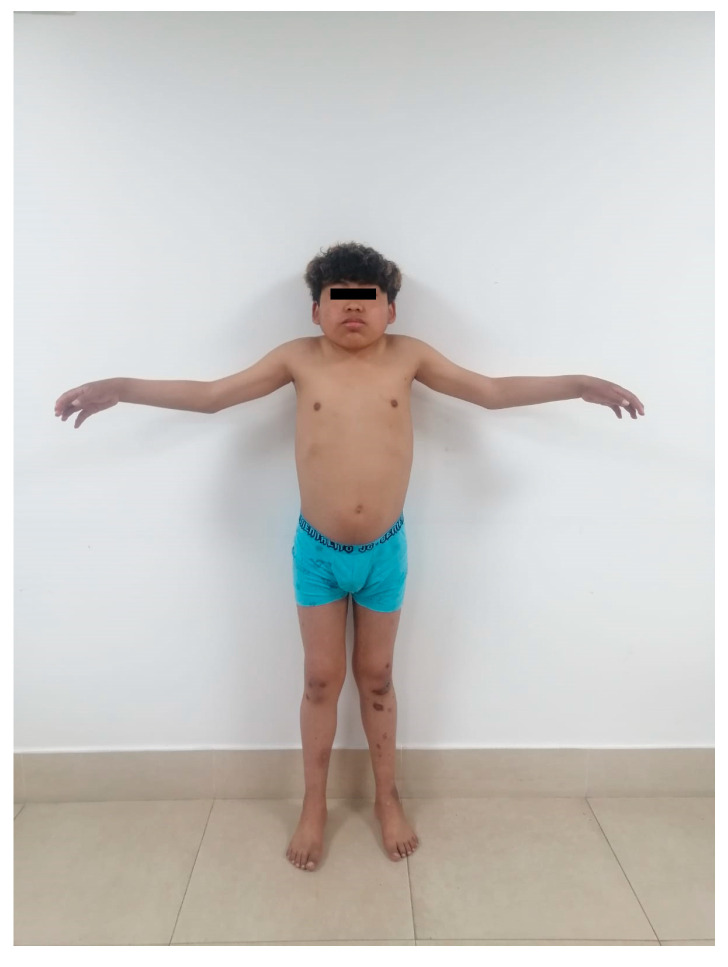
Short stature, low hair implantation, hypertelorism, short neck.

We describe a seventeen-year-old male patient, who is a product of a fourth uncomplicated pregnancy, with a paternal history of type 2 diabetes. He presented with a slowing of growth rate from the age of 5 years and hyperglycemia without ketoacidosis at 8 years, when he was diagnosed with type 1 diabetes and was started on intermediate acting insulin NPH with poor adherence. At age 17, he came to the emergency room with a 2 month history of facial and lower limb edema. Upon physical examination, his vital signs were: 99/60 mmHg, heart rate 110 bpm, and respiratory rate 22 rpm. His weight was 34 Kg (<3rd percentile), height 135 cm (<3rd percentile), body mass index 18.6 kg/m^2^ (10th percentile), arm span 126 cm, upper segment 64 cm, lower segment 71 cm, and upper/lower segment ratio 0.90. The patient was awake, with dysmorphic features (low hair implantation, hypertelorism, epicanthal fold, short neck), Tanner II, palpebral edema bilaterally, and edema in lower limbs ++/++++ (Figure 1).

**Figure 2:**
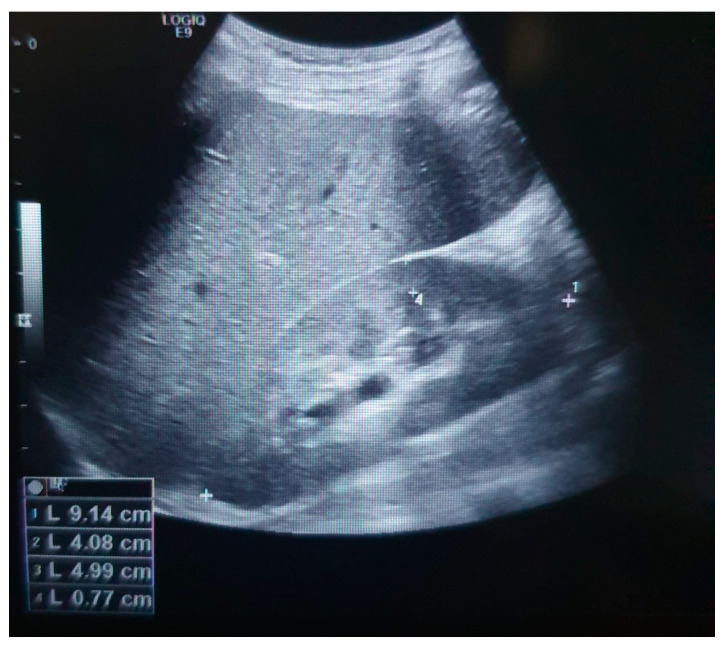
Renal ultrasound showing mild pyelocalyceal dilation.

**Figure 3:**
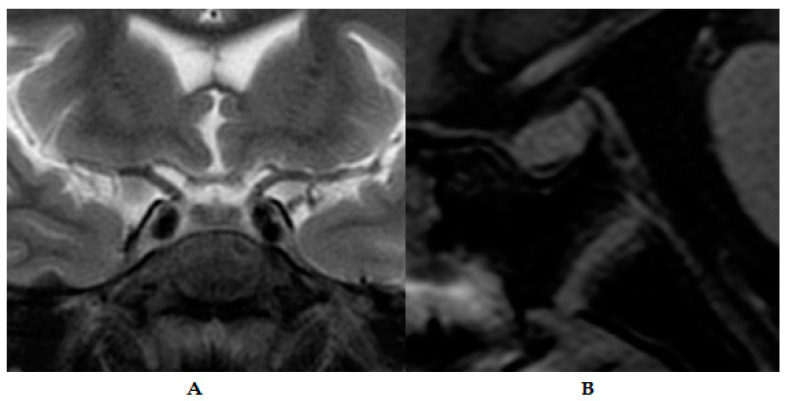
Contrasted magnetic resonance imaging (MRI) of pituitary gland. (**A**) Coronal view. (**B**) Sagittal view.

Biochemical examination showed a fasting glucose of 472.50 mg/dL (60–100 mg/dL), HbA1C (Hemoglobin A1C) of 18.66% (4.8–5.90%), and detectable C peptide 0.64 ng/mL (1.10–4.40 ng/mL). Ketones were negative. Glutamic acid decarboxylase (GAD) and tyrosine phosphatase islet antigen-2 autoantibodies were negative. He also had glomerular hyperfiltration CKD-EPI 188 mL/min/1.73 m^2^ and proteinuria close to nephrotic range 2902 mg/24 h (normal value < 140 mg/24 h). Renal ultrasound showed mild pyelocalyceal dilation (Figure 2). Liver function was normal. Basal pituitary hormone levels were: IGF-1 62 ng/mL (190–429 ng/mL), testosterone <0.025 ng/mL (1.93–7.40 ng/mL), LH 0.54 mUI/mL (1.7–8.6 mUI/mL), FSH 0.64 mUI/mL (1.5–12.4 mUI/mL), TSH 3.12 uIU/mL (0.27–4.20 uIU/mL), FT4 1.03 ng/dL (0.93–1.76 ng/dL), and prolactin 6.29 ng/mL (4.04–15.20 ng/mL). A growth hormone (GH) clonidine stimulation test was performed, with the following results: baseline 3.72 ng/mL, 60 min 10.09 ng/mL, 90 min 5.06 ng/mL, 120 min 7.24 ng/mL, and 180 min 7.79 ng/mL. Pituitary MRI with contrast revealed no apparent lesions (Figure 3). Bone age (Greulich–Pyle) was 11 years 6 months. Peripheral blood karyotype was male 46, XY. MODY probability calculator score could not be calculated because his HbA1C was higher than 15%. Dilated fundus examination (DFE) revealed proliferative DR and cataracts.

Whole exome sequencing (WES) study was performed using saliva DNA and Illumina technology where a missense variant in the insulin gene c94G>A (p.Gly32Ser) was identified, according to Clinvar corresponding to MODY10 (OMIM #613370), hyperproinsulinemia (OMIN #616214), and neonatal diabetes mellitus (OMIN #618858). Sanger sequencing was negative in samples of the patient’s first degree relatives, assuming “de novo” variant in the family. No other findings were present in the WES.

The patient was treated with basal insulin glargine at bedtime, and prandial insulin regular (titrated to total daily dose 1/UI/kg/day) plus empagliflozin 25 mg orally daily. After 3 years of regular follow-up, A1c dropped from 18.66% to 8.6%, and proteinuria decreased from 2902 mg/24 h to 1082.65 mg/24 h (60% decrease); puberty resumed, and the patient achieved Tanner Stage IV and a final height of 146 cm (11 cm gain).

## 3. Discussion

Given its low frequency, the possibility of MD is often not considered in people with diabetes. However, certain factors can make one suspect that the diagnosis of type 1 or type 2 diabetes is not accurate. A combination of tests and clinical factors help rule out type 1 or type 2 diabetes and identify the MODY. Likewise, factors taken into account and that may indicate MD are: the presence of other disorders caused by a specific genetic mutation (e.g., a cyst in the kidneys), absence of obesity or diabetic relatives of normal weight, diagnosis of diabetes during the first 6 months of life, ethnic origin (e.g., white people of European roots have a lesser prevalence of type 2 diabetes), and family history of diabetes (especially when it affects one of the parents). Nonetheless, none of these factors alone allow for the diagnosis of MD. Therefore, it is crucial to consider them globally along with blood test results. Resolution through genetic testing can determine if the case has an MODY-causing gene [13]. Testing relatives of a patient with MODY can help detect the presence or risk of diabetes.

**Figure 4:**
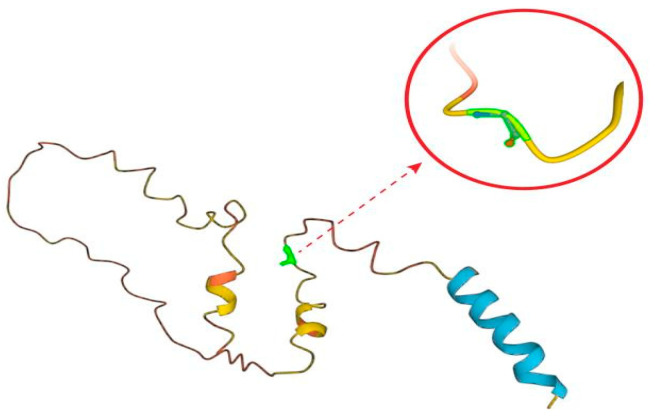
Pathogenic heterozygous variant p.Gly32Ser of the insulin protein that attenuates secretion and affects disulfide pairing.

Our diabetes mellitus case had an unusual presentation, as the disease started at an early age, had negative autoantibodies with severe hyperglycemia, and atypical features without ketoacidosis. This discordant pattern for type 1 or type 2 DM led us to additional genetic testing that led to the diagnosis of MD. The Exeter diabetes MODY probability calculator (htpps://diabetesgenes.org/exter-diabetes-app/ModyCalculator) (accessed on 4 July 2023) [14] could not be utilized due to severe hyperglycemia exceeding the upper limit of the calculator because it was validated for less severe forms of MODY, such as MODY1, MODY2, and MODY3. Gene testing showed a heterozygous pathogenic variant of the INS gene c94G>A (p.Gly32Ser) located in exon 2, position 111 of codon 204, that has previously been reported to be pathogenic. INS encodes preproinsulin, which includes 110 amino acids, and then preproinsulin forms proinsulin though cleavage of the signal peptide in the reticulum in beta cells. Furthermore, the folding action of proinsulin is managed, and three native disulfide bonds (A6-A11, B7-A7, and B19-A20) are formed before the cleaving of C-peptide. About 70 mutations in the insulin gene have been recognized [15]. Specifically, p.Gly32Ser mutation has been linked to permanent neonatal DM, albeit that variable ages of onset have also been observed [16,17]. As this mutation occurs at the conserved B8 glycine, close to the B7 cysteine, it affects disulfide bond formation, causing the misfolding of preproinsulin, and partial retention in the endoplasmic reticulum (ER), triggering ER stress and ß cell death [17] (Figure 4).

Clinical manifestations in MODY10 patients are variable as mildly affected patients can be controlled with diet and exercise, or in more severe cases as this patient, the clinical course is similar to that of patients with type 1 diabetes in the progressive beta cell deficiency requiring insulin therapy for life [18,19]. There are few reports of microvascular complications with MODY10, with most cases presenting with retinal (mild or severe retinopathy) and kidney involvement (microalbuminuria or DN), and neuropathy [20]. Our patient showed glomerular hyperfiltration and proteinuria with renal ultrasound showing mild pyelocalyceal dilation. Hyperfiltration is associated with the development and progression of DN in type 1 and 2 diabetes mellitus patients [21,22]. The benefit of sodium-glucose cotransporter 2 inhibitors (SGLT2i) in type 1 and 2 diabetes patients with micro and macro albuminuria is well established [23,24], as these drugs reduce proteinuria and prevent progression of CKD [25,26]. At this stage, there are few data regarding the use of this class of drugs in other types of MODY [27], and have not been reported its use in MODY10 patients. At 3 years of follow up and after titration of insulin and empagliflozin doses, a reduction in proteinuria was observed. DR was managed with anti-vascular endothelial growth factor (VEGF) injections, phacoemulsification and intraocular lens implantation, posterior vitrectomy, and endolaser photocoagulation.

Delayed puberty and retardation of growth were observed in this patient. In the WES study, no pathogenic or probably pathogenic variants were identified in genes related to hypogonadism and short stature that could explain the patient’s clinical condition. It is known that severe hyperglycemia and chronic inflammation in poorly controlled type 1 diabetes mellitus produces poor lineal growth. This is thought to be due to low IGF-1 levels caused by the upregulated expression of insulin-like growth factor-binding protein (IGFBP-1) secondary to low portal insulin; this is the most likely mechanism of disease in our patient given his severe degree of hyperglycemia [28,29]. On the other hand, as of the date of writing this report, researchers have not found any previous descriptions of functional hypogonadotropic hypogonadism in association with insulin gene mutation, and that it is probably caused by metabolic endotoxemia as in other types of diabetes mellitus [30]. Also, a catabolic state characterized by low levels of insulin and leptin inhibits the expression of kisspeptin in the central nervous system. Kisspeptin plays a crucial role as a stimulator of GnRH production, and its deficiency represents another plausible mechanism contributing to functional hypogonadism [31].

The diagnosis of this type of diabetes includes a series of clinical, biochemical, and genetic characteristics that may not be entirely present [13]. The advancement of molecular genetics has allowed a better classification and diagnosis of MODY. However, in many cases, subjects with this type of diabetes are misdiagnosed as having diabetes type 1 (10%) or type 2 (2–5%). For the diagnosis, a high index of suspicion is needed, and it is essential to consider family history, age of onset, the degree of hyperglycemia, and the absence of pancreatic autoantibodies [32]. Three main barriers for an early and accurate diagnosis of MODY have been described [33]: patient-related issues (lack of reliable personal and family history, loss to follow-up, personality issues), physician-related issues (deficient knowledge and awareness related to different types of diabetes mellitus besides type 1 and type 2 DM), and healthcare system-related issues (cost of testing, insufficient number of knowledgeable healthcare providers, poor education and support resources). Most of the misdiagnoses and delays in treatment are related to a lack of knowledge from healthcare providers. However, in 15–20% of clinically compatible cases with a diagnosis of MODY, no genetic cause is found (those so far called MODY-X), suggesting the existence of other genes involved in this disease that can condition the emergence of new subtypes soon.

Therefore, the importance of a correct diagnosis is essential to avoid erroneous treatments. Likewise, this can be useful to correctly classify the parents and, if proper, receive the right treatment. Establishing the clinical diagnosis of MODY and the molecular diagnosis of MODY subtypes is very important for appropriate patient care. It is essential to note that the diagnosis of MODY should be suspected in a broader range of clinical situations than initially described [34]. Further research is needed to more precisely define the phenotypic characteristics and clinical markers that facilitate the finding of a positive molecular diagnosis for MODY subtypes. Clinically, MODY is diabetes with a very heterogeneous severity, even among members of the same family. Finally, it should be noted that although mutations in INS generally cause neonatal diabetes, as has been presented, they can also lead to MODY diabetes in adolescents and adults.

## 4. Conclusions

MD is rare and MODY10 is among the less frequent types. Given its unknown prevalence in Latin American countries, case reports such as this are valuable. MODY should be suspected in patients with type 1 phenotype with negative autoimmunity even in the absence of a family history of diabetes. In our patient, inadequate metabolic control and late diagnosis led to the development of complications. Efforts must be made to increase awareness of MODY amongst health professionals and to ensure widespread access to proper diagnostic tests and treatment modalities for MODY patients. Our experience highlights the potential synergistic benefit of SGLT2i and insulin in proteinuria reduction and improvement of glycemic control on MODY10 patients, that should be explored in long-term clinical studies. Functional hypogonadotropic hypogonadism reverted after improvement of glucotoxicity.

## Data Availability

Data are contained within the article.
